# A Chinese Pane-Like 2D Metal-Organic Framework Showing Magnetic Relaxation and Luminescence Dual-Functions

**DOI:** 10.1038/s41598-017-11006-5

**Published:** 2017-09-11

**Authors:** Cai-Ming Liu, De-Qing Zhang, Xiang Hao, Dao-Ben Zhu

**Affiliations:** Beijing National Laboratory for Molecular Sciences, Center for Molecular Science, Key Laboratory of Organic Solids, Institute of Chemistry, Chinese Academy of Sciences, No.2 1st North Street, Zhongguancun, Beijing, 100190 P.R. China

## Abstract

The discovery of graphene kicked off the curtain of atom-type two-dimensional (2D) materials. Layered metal-organic frameworks (MOFs) as parallel molecule-based 2D materials are more designable and more diverse, and magnetism may be induced by their metal ion nodes. However, the multifunctional 2D plane-like MOFs are very difficult to obtain. Here we describe a Chinese pane-like 2D MOF constructed from the Ln^3+^ cation and the nanoscale luminescent tritopic ligand tris(4′-carboxybiphenyl)-amine, responding to the slow magnetic relaxation and luminescence properties, respectively. Notably, the Dy-Dy distances separated by the tritopic ligand are up to 2 nm. Such a 2D molecular material is expected to have potential applications in optoelectronics and multimodal sensing.

## Introduction

Two-dimensional (2D) materials have become most exciting and popular multifunctional materials since the discovery of graphene in 2004^1^. Functional layered metal-organic frameworks (MOFs) can be viewed as molecule-based 2D materials^2–4^, whose pronounced advantages are more designable and more diverse, and their metal ion knots may induce good magnetism. Nevertheless, it is an extreme challenge to design the MOF-type 2D materials with multifunctions similar to the atom-type 2D materials. Recently, lanthanide(III) ions, especially the Dy(III) ion, which contain unique 4f orbital electrons showing strong spin-orbit coupling and high magnetic moment, have been chosen as the metal nodes to construct lanthanide-based MOFs (LnMOFs), endowing single-molecule magnet (SMM) behaviors^5–7^. The SMMs feature magnetic hysteresis and slow magnetic relaxation at low temperatures, with potential applications in magnetic devices for high-density information storage, quantum computing and spintronics^8–14^. The lanthanide (III) ions, each of which is equivalent to a single-ion magnet due to very weak magnetic exchange interactions between the lanthanide (III) ions containing shielded 4f electrons, may be arranged into highly ordered nodes with nanoscale-separation in the LnMOFs; such materials are convenient for assembly of corresponding molecular magnetic devices. Notably, the 2D layered LnMOF systems behaving as SMMs are especially attractive for this objective^15^.

**Figure 1:**
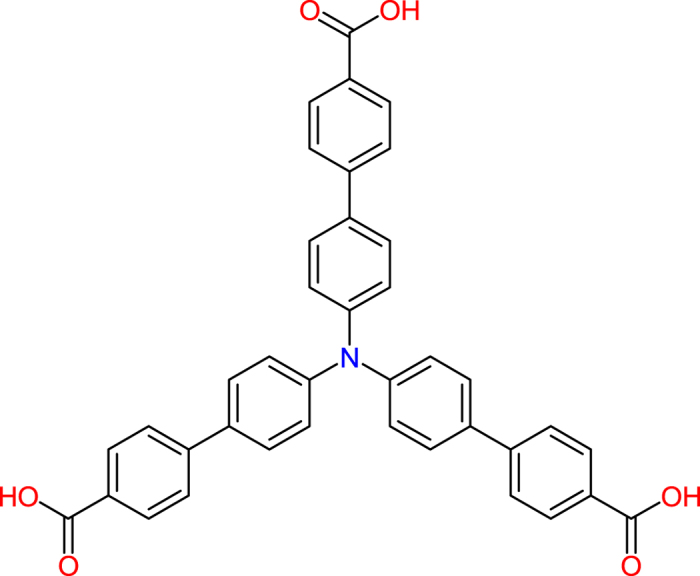
Molecular structure of H_3_TCBPA.

Although some LnMOFs themselves may display photoluminescence by the aid of ‘antenna effect’ of ligands^16^, not all of LnMOFs are luminescent because the photoluminescence of lanthanide ions is subject to the energy’s match rule. Alternatively, using a fluorescent spacer ligand to assemble LnMOFs should be an effective approach to the optical-magnetic multifunctional materials, this strategy allows both the SMM and luminescence properties from different building blocks to be combined together within a MOF system, guaranteeing both superparamagnet and emission functions. The motivation to explore the emissive quantum magnets is that they are potentially applicable in optoelectronics and multimodal sensing^17^. However, the reported luminescent SMMs are limited, which concentrate on mononuclear^17–21^, polynuclear^22–26^, and chainlike systems^27^, the development of high-dimensional luminescent Ln(III) complexes showing SMM properties is still at a primitive stage^28^; notably, the photoluminescence in these molecular materials mainly originates from the lanthanide(III) ions, the case with fluorescent bridging ligands has not been explored. Recently, we have adopted fluorescent tris(4′-carboxybiphenyl)-amine^29^ (H_3_TCBPA, Fig. 1) as the spacer to construct multifunctional LnMOF-type 2D materials. Herein, we describe the solvothermal synthesis, crystal structure, luminescent and magnetic properties of a 2D LnMOF, [Dy(TCBPA)(H_2_O)_2_]_n_· guest (guest: 2DMF·4H_2_O, **1**). Remarkably, **1** possesses an interesting plane-like network structure similar to one classical Chinese pane made of parallelograms, exhibiting not only slow magnetic relaxation responded to the Ln^3+^ knot but also ligand-based luminescence. Its erbium(III) analogue [Er(TCBPA)(H_2_O)_2_]_n_· guest (guest: 2DMF·4H_2_O, **2**) is reported too.

## Results and Discussion

### Preparation

Pale yellow crystals of **1** or **2** were obtained as the only crystalline phase through solvothermal reaction between tris(4′-carboxybiphenyl)amine and Ln(NO_3_)_3_ ·5H_2_O in an DMF/H_2_O solution in the presence of 2-fluorobenzoic acid, which just serves as a directing agent and/or a modulator^30^. It is noteworthy that this and other nanoscale tritopic ligands are inclined to construct 3D LnMOFs, acting as the linkers^31–36^, whereas we successfully obtained the 2D layered LnMOFs with nanoscale tritopic ligands for the first time.

### Structural description

**Figure 2:**
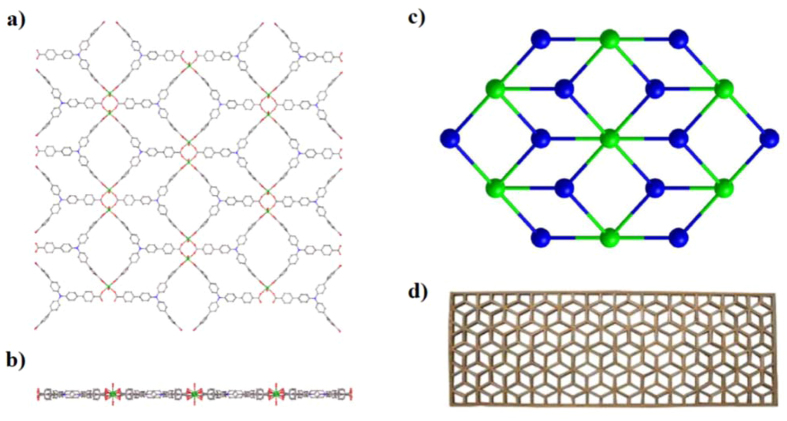
Top view (**a**) and side elevation (**b**) of the 2D plane-like network of **1**; the topological structure of **1**(**c**), in which the Dy(III)_2_ dinuclear cluster and the TCBPA^3−^ ligand act as the 6-connected and 3-connected vertices, respectively; one classical Chinese pane made of parallelograms (**d**), which has the same topology structure as **1**.

Complex **1** crystallizes in the monoclinic space group *C*_2/m_, its structure exhibits a 2D plane-like network, which is constructed from Dy(III)_2_ dinuclear cluster nodes, TCBPA^3−^ bridging ligands and water terminal ligands (Fig. 2). The Dy atom is coordinated by two water O atoms and six carboxylate O atoms from four TCBPA^3−^ ligands. This [DyO_8_] eight-coordination geometry was ascertained to be the biaugmented trigonal prism by the Shape software^37^, and the calculation indicates that the deviation value from this polyhedron’s ideal *C*_2v_ symmetry is 3.321 (Table S1, SI). Each TCBPA^3−^ ligand bridges four Dy^3+^ ions *via* three carboxylate groups (Fig. 2a): two of which coordinate to two discrete Dy atoms with the chelating mode; whereas the third one links to two neighbouring Dy atoms with the *syn*–*syn* bridging mode, and the bridged Dy-Dy separation is 5.155 Å. Notably, the Dy-Dy distances separated by the TCBPA^3−^ bridging ligand are 21.555 Å, 21.555 Å and 19.641 Å,respectively. From a topological view, the Dy(III)_2_ dinuclear cluster can be considered as a 6-connected node and the TCBPA^3−^ bridging ligand as a 3-connected node, therefore a simplified (3, 6)-connected network is generated (Fig. 2c), whose long topological (O’Keeffe) vertex symbol is 4.4.4.4.4.4.6.6.6.6.6.6 and the short (Schläfli) vertex symbol 4^6^.6^6^.8^3^, according to the analytical results by the Olex program. Such an interesting plane topology is reminiscent of one classical Chinese pane made of parallelograms (Fig. 2d). Complex **2** is of the same structure as **1**, but using Er instead of Dy (Fig. S1, SI). The average Dy-O bond distance of **1** is 2.357 Å (Table S2, SI), obviously larger than the mean Er-O bond distance of **2** (2.338 Å), being ascribed to the lanthanide contraction effect. Clearly, such a plane-like structure is totally different from those of the 3D LnMOFs based on the same ligand^31^.

### Magnetic properties

**Figure 3:**
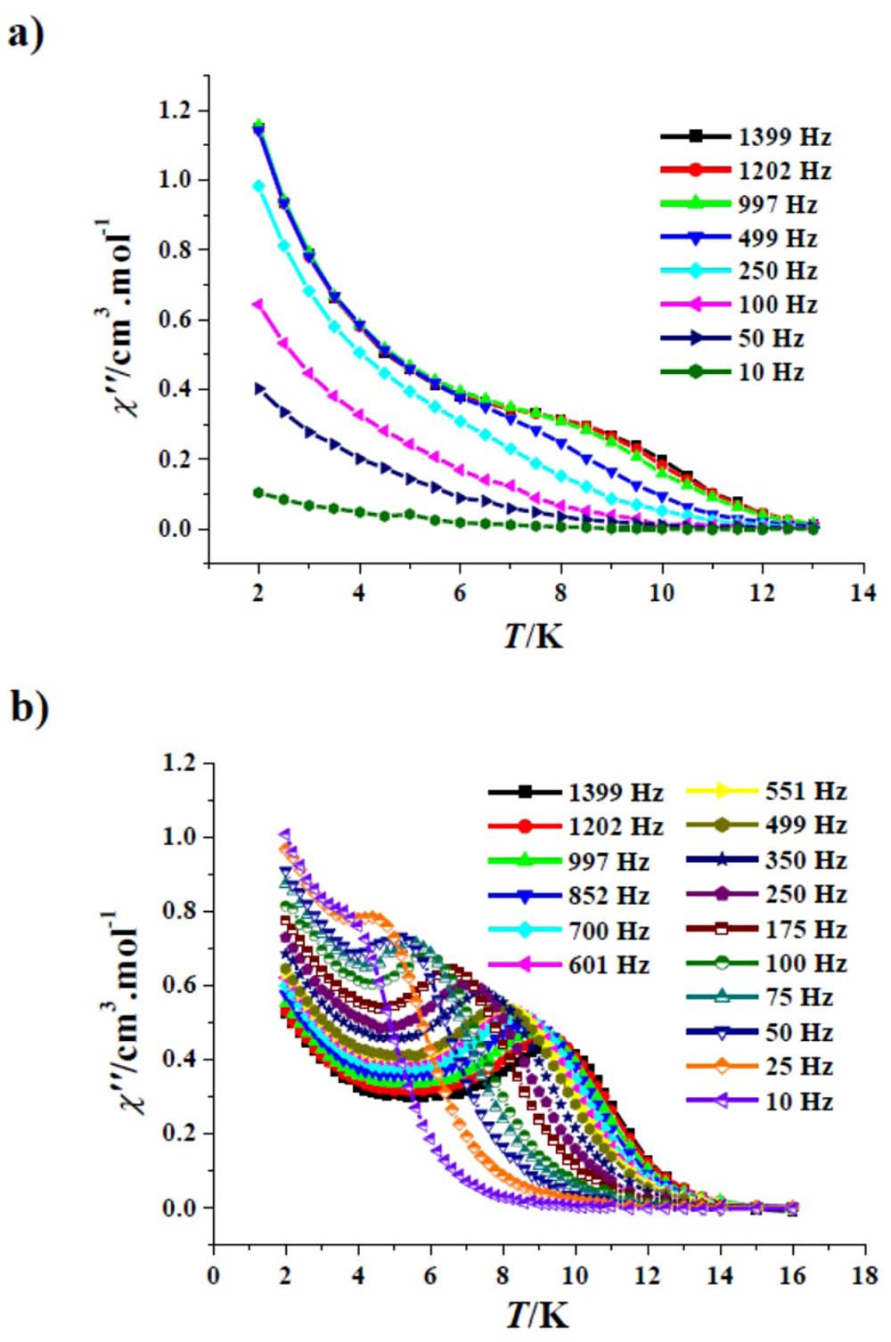
Plots of *χ*″ *vs T* for **1** (*H*_dc_ = 0 Oe, *H*_ac_ = 2.5 Oe) (**a**) and (*H*_dc_ = 1 kOe, *H*_ac_ = 2.5 Oe) (**b**).

The temperature dependence of magnetic susceptibility for complex **1** was investigated under 1 kOe in the range of 2–300 K. The room temperature *χT* value of 14.15 cm^3^ K mol^−1^ for **1** is in agreement with the value of 14.17 cm^3^ K mol^−1^ for one isolated Dy^3+^ ion in the ^6^ *H*_15/2_ state (Fig. S2, SI). The *χT* product declines gently with a temperature decrease in the range of 300–50 K, then falls steeply to reach 10.60 cm^3^ K mol^−1^ at 2 K, which mainly originates from thermal depopulation of the excited Stark sublevels for the Dy^3+^ ions. The Curie-Weiss equation was used to fit magnetic susceptibility data, giving the *C* value of 14.27 cm^3^ K mol^−1^ and the *θ* value of −1.50 K; this very small negative *θ* value suggests that the possible dipole–dipole antiferromagnetic interactions between the Dy^3+^ ions is negligible. The field-dependent magnetization measured at 2–6 K revealed that there exists magnetic anisotropy in **1** as the *M vs H*/*T* plots are non-superimposed (Fig. S3, SI). Therefore, alternating-current (ac) susceptibility measurements as a function of temperature were performed to explore the dynamics of the magnetization of **1**. Besides the frequency dependence of the out-of-phase (*χ*′) (Fig. S4, SI), the temperature dependence of the out-of-phase (*χ″* ) determined in zero dc field shows significant signals below 12 K (Fig. 3a), indicating existence of SMM behaviours. However, no peaks could be detected owing to the obvious quantum-tunnelling effects. A dc field generally may suppress such quantum-tunnelling effects^38–40^. The field-dependent *χ″* measured at 250 Hz and 7 K (Fig. S5, SI) indicates that the *χ″* signals are enhanced with increased fields, but the change is small when the dc field is larger than 1 kOe. Therefore, a 1 kOe dc field was applied to overcome the quantum-tunnelling effects, and not only *χ*′ (Fig. S6, SI) but also *χ″* (Fig. 3b) peaks actually appeared at frequencies more than 25 Hz.

In order to assess the effective barrier for the magnetic relaxation process of **1**, the frequency-dependent *χ″* peak temperature, in the format of the ln(*τ*) *vs* 1/*T* plot, was fitted to the Arrhenius law, *τ* = *τ*_0_exp(*U*_eff_/*kT*), affording *U*_eff_/*k* value of 70.1(1.1) K and *τ*_0_ value of 6.6(0.1) × 10^−8^ s (Fig. S7, SI). The *τ*_0_ value is normal for the SMMs/SIMs (10^−5^–10^−11^ s). The *U*_eff_/*k* value of **1** is at a moderate level of carboxylate containing complexes^41^. The SMM behaviours of **1** were also supported by the parameter *Φ* {= (Δ*T*_f_/*T*_f_)/Δ(log*f* )} calculation^42^, where *T*_f_ is the *χ″* peak temperature, and *f* the frequency; the *Φ* value of 0.29 for **1** is actually in line with the superparamagnet value (*Φ* > 0.1), but much larger than the spin glass value (*Φ* ≈ 0.01)^42^. Furthermore, under a dc field of 1 kOe, the frequency-dependent *χ″* signals of **1** display well-defined temperature-dependent peaks at 4–9 K, being indicative of slow magnetic relaxation too (Fig. S8, SI). At 7 K and 8 K, two classical semicircle Cole-Cole diagrams were observed (*χ″ vs χ*′, Fig. S9, SI), which could be fitted by a Debye model for a single relaxation time (*τ*)^43, 44^, giving *α* values of 0.096 and 0.066 for 7 and 8 K, respectively. The small *α* values suggest narrow distributions of relaxation times. Furthermore, no loop was observed for **1** at 1.9 K (Fig. S10, SI). Based on the very weak magnetic interactions among the Dy^3+^ cations and the low symmetry of the Dy^3+^ cation, we assume that a Ising ground state of the Dy^3+^ cation exists in **1**, the electrostatic model^45^ was therefore used to estimate the magnetic axis of the dysprosium(III) ion. As shown in Fig. S11 (SI), the calculated magnetic axis directionality is very close to the line defined by Dy1-C13, with a small angle of 2.48°.

The magnetic relaxation of complex **2** was also studied for comparison. The *χT* value of 14.46 cm^3^ K mol^−1^ at room temperature for **2** is concordant with the theory value of 11.48 cm^3^ K mol^−1^ for one Er^3+^ ion (^6^ *I*_15/2_, Fig. S12, SI). Unlike complex **1**, the erbium(III) analogue **2** didn't display any obvious *χ*″ signals when the dc field was zero (Fig. S13, SI). Although the 2 kOe dc field can let complex **2** present both frequency-dependent *χ*′ signals (Fig. S14, SI) and frequency-dependent *χ*″ signals (Fig. S15, SI), no peaks could be observed in the *χ*″ curves even at 1399 Hz and at above 2.0 K, which suggest a very small effective energy barrier value that could not be calculated in the above mentioned way based on the ln(*τ*) *vs* 1/*T* plot. This result indicates that the biaugmented trigonal prism of the [ErO_8_] coordination geometry is not beneficial to present SMM behaviors^46^.

### Luminescence

**Figure 4:**
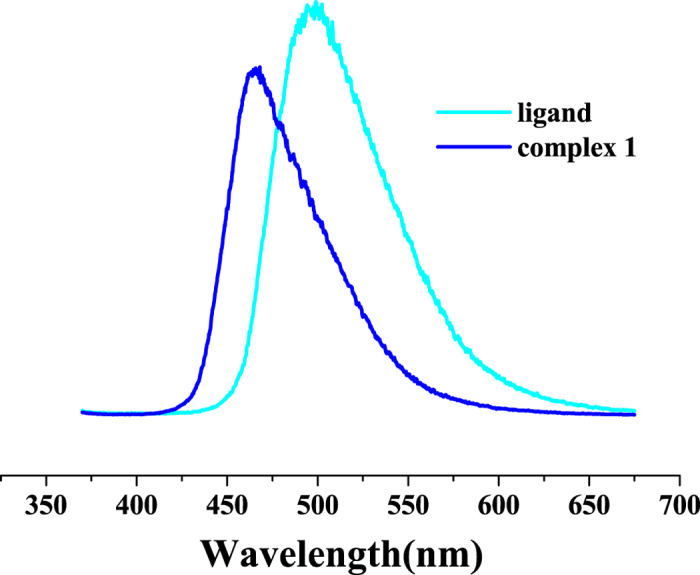
Solid-state emission spectra of **1** and free ligand at room temperature (*λ*_ex_ = 350 nm).

The typical narrow luminescence bands arising from the Ln^3+^ cation are absent in the solid state photoluminescence spectrum of **1** at room temperature (Fig. 4). Nevertheless, upon excitation with 350 nm UV light, complex **1** displays a broad emission band at 465 nm, which is similar to the large emission band at 498 nm for the free H_3_L ligand due to the *π*-π* transition, but a 33 nm of blue-shift was observed. Obviously, the ligand’s coordination to the metal ion induces the luminescence’s blue-shift^31^. The average fluorescence lifetime of **1** was determined to be 0.79 ns (*λ*_ex_ = 360 nm, Fig. S16, SI), shorter than 2.06 ns (*λ*_ex_ = 360 nm, Fig. S17, SI) of the free ligand. Furthermore, complex **1** shows a smaller absolute emission quantum yield (2.69%) than the free ligand (24.32%) (*λ*_ex_ = 360 nm), which can be mainly attributed to the solvent effect because **1** contains coordinated water molecules and some solvent molecules within the crystalline host lattices.

Similar to **1**, only ligand-based luminescence spectrum could be observed for **2** in the solid state at room temperature. A broad emission band at 462 nm was excited by 360 nm UV light, which has a 36 nm of blue-shift comparing to free H_3_L ligand because of the ligand to metal coordination role again (Fig. S18, SI). The absolute emission quantum yield (1.11%) of **2** is smaller than that of **1**, and the fluorescence lifetime of **2** is too fast (<0.7 ns) to run lifetime measurement with the Edinburgh Analytical Instruments FLS980.

## Conclusions

In summary, we present the first 2D layered LnMOF based on nanoscale tritopic ligands, which possesses an interesting plane-like structure and shows both slow magnetic relaxation and luminescence properties. We demonstrate a promising approach to the multifunctional MOFs using the magnetic Ln^3+^ cation and the luminescent bridging ligand as the node and the linker, respectively. Our work also opens up a designable avenue to novel MOF-type 2D materials with interesting optical/magnetic multifunctions, such molecule-based 2D materials are expected to have potential applications in many fields such as optoelectronics and multimodal sensing.

## Methods

### Materials and instrumentation

Tris(4′-carboxybiphenyl)amine was synthesized following reported procedures^29^. Other chemicals and solvents were obtained commercially and used as received. The elemental analyses (C, H, N) were accomplished on a Vario ELIII elemental analyser. The FT-IR spectra were determined on a Bruker/Tensor-27 spectrophotometer with pressed KBr pellets in the range 4000–400 cm^−1^. The fluorescence spectra, the fluorescence lifetime and the absolute emission quantum yield were measured on an Edinburgh Analytical Instruments FLS980. Both the dc and ac magnetic susceptibility measurements were carried out on a Quantum Design MPMS-XL5 SQUID magnetometer using Pascal’s constants diamagnetic corrections.

### Preparation of 1 and 2

A mixture of tris(4′-carboxybiphenyl)amine (0.05 mmol), 2-fluorobenzoic acid (1.0 mmol), Ln(NO_3_)_3_ ·5H_2_O (0.05 mmol), DMF (2.5 mL) and H_2_O (0.75 mL) in a 25 mL Teflonlined stainless steel autoclave was maintained at 105 °C for 3 days. After the autoclave had been cooled slowly to 20 °C during 10 hours, light yellow plate crystals of **1** (Ln = Dy, 65% yield based on Dy), or light yellow plate crystals of **2** (Ln = Er, 58% yield based on Er) were harvested. These crystals were washed with water and dried at ambient temperature in air. Anal. Calcd (%) for C_45_H_50_DyN_3_O_14_ (**1**): C 53.02; H 4.94; N 4.12. Found: C 53.06; H 4.97; N 4.09. IR (KBr, cm^−1^): 3443(b, s), 3033(w), 2928(w), 1663(m), 1600(m), 1522(m), 1492(w), 1424(s), 1324(w), 1280(w), 1188(w), 1112(w), 1088(w), 862(w), 835(w), 788(m), 731(w), 708(w), 685(w), 656(w), 576(w), 558(w), 475(w), 429(w). Anal. Calcd (%) for C_45_H_50_ErN_3_O_14_ (**2**): C 52.77; H 4.92; N 4.10. Found: C 52.73; H 4.95; N 4.08. IR (KBr, cm^−1^): 3401(b, s), 3033(w), 2932(w), 1666(m), 1599(m), 1523(m), 1493(w), 1426(s), 1324(w), 1282(w), 1188(w), 1111(w), 1089(w), 864(w), 835(w), 788(m), 732(w), 709(w), 685(w), 656(w), 577(w), 560(w), 477(w), 429(w).

### X-ray collection and structure determination

A single crystal with dimensions 0.14 × 0.11 × 0.06 mm^3^ of **1** or 0.26 × 0.09 × 0.06 mm^3^ of **2** was chosen to collect data on a Bruker SMART APEX-CCD diffractometer with Mo-K_*α*_ radiation (*λ* = 0.71073 Å) at 173(2) K. Empirical absorption corrections from *φ* scan were applied. Cell parameters were obtained by the global refinement of the positions of all collected reflections for two complexes. The structures were solved by direct methods and refined by a full matrix least-squares technique based on *F* ^2^ using with the SHELX-2014 program package. The DMF and H_2_O solvent molecules in the complexes are highly disordered. The SQUEEZE subroutine of the PLATON software suit was used to remove the scattering from the highly disordered guest molecules. All non-hydrogen atoms were refined anisotropically, and all hydrogen atoms except those in coordinated water molecules were refined as riding atoms. Crystallographic data and structure determination parameters of complexes **1** and **2** are given in Table S3. CCDC-1557227 (**1**) and 1557228 (**2**) contain the supplementary crystallographic data, which can be obtained free of charge from The Cambridge Crystallographic Data Centre via www.ccdc.cam.ac.uk/data_request/cif.

## Electronic supplementary material

A Chinese Windowpane-Like 2D Metal-Organic Framework Showing Magnetic Relaxation and Luminescence Dual-Functions
