# Supplementary material for: A Chinese Pane-Like 2D Metal-Organic Framework Showing Magnetic Relaxation and Luminescence Dual-Functions
Source: Sci Rep. 2017 Sep 11;7:11156. doi: 10.1038/s41598-017-11006-5 (PMC5594028; doi:10.1038/s41598-017-11006-5)
Supplement: Supplementary file 1 — A Chinese Windowpane-Like 2D Metal-Organic Framework Showing Magnetic Relaxation and Luminescence Dual-Functions [file 41598_2017_11006_MOESM1_ESM.pdf]

A Chinese Windowpane-Like 2D Metal-Organic Framework Showing Magnetic Relaxation and Luminescence Dual-Functions

Cai-Ming Liu,*[1] De-Qing Zhang,*[1] Xiang Hao,[1] and Dao-Ben Zhu[1]

[1] *Beijing National Laboratory for Molecular Sciences, Center for Molecular Science, Key Laboratory of Organic Solids, Institute of Chemistry, Chinese Academy of Sciences, Zhongguancun North 1 Street No. 2, Beijing 100190, P. R. China*

Table S1. Continuous Shape Measures calculation for the Dy(III) ion in 1.

Dy1, eight-coordination

OP-8	1 D8h	Octagon
HPY-8	2 C7v	Heptagonal pyramid
HBPY-8	3 D6h	Hexagonal bipyramid
CU-8	4 Oh	Cube
SAPR-8	5 D4d	Square antiprism
TDD-8	6 D2d	Triangular dodecahedron
JGBF-8	7 D2d	Johnson gyrobifastigium J26
JETBPY-8	8 D3h	Johnson elongated triangular bipyramid J14
JBTPR-8	9 C2v	Biaugmented trigonal prism J50
BTPR-8	10 C2v	Biaugmented trigonal prism
JSD-8	11 D2d	Snub diphenoid J84
TT-8	12 Td	Triakis tetrahedron
ETBPY-8	13 D3h	Elongated trigonal bipyramid

**Structure**	**OP-8**	**HPY-8**	**HBPY-8**	**CU-8**	**SAPR-8**	**TDD-8**	**J GBF-8**	**JETBPY-8**	**JBTPR-8**	**BTPR-8**	**JSD-8**	**TT-8**	**ETBPY-8**
**ABOXIY,**	**32.562,**	**21.449,**	**17.944,**	**14.062,**	**4.377,**	**3.564,**	**14.547,**	**28.512,**	**3.680,**	**3.321,**	**5.446,**	**14.368,**	**24.546**

a)

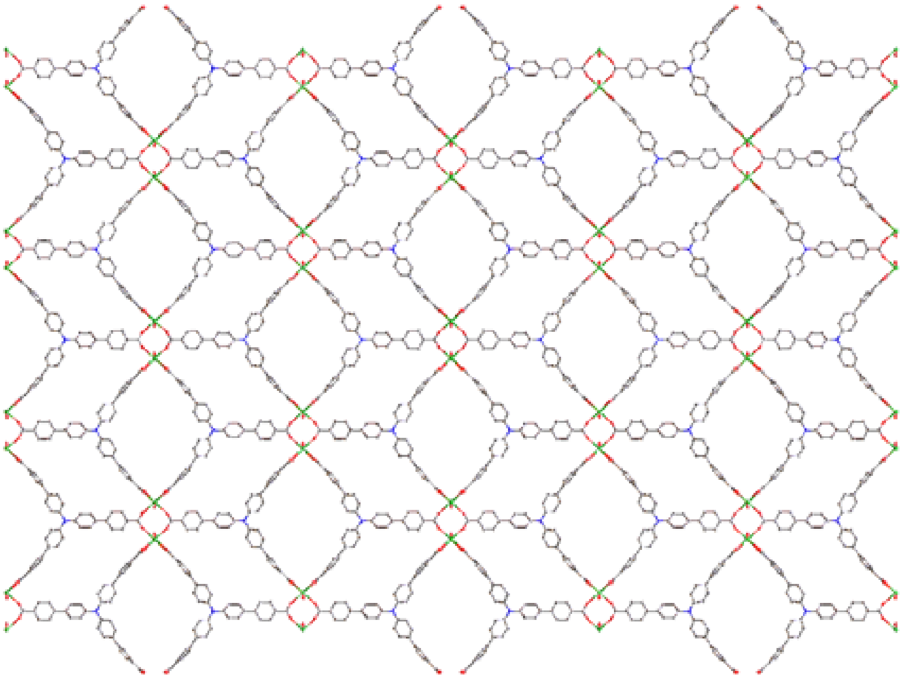

b)

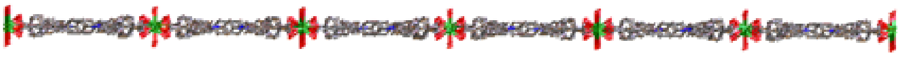

Fig. S1. Top view (a) and side elevation (b) of the 2D plane network of **2**.

Table S2. Selected Bond Distances(Å) and Angles (°) for 1 and 2.

		**1**	
Dy1-O3$^{\#1}$	2.232(4)	Dy1-O3	2.232(4)
Dy1-O1W	2.390(10)	Dy1-O2W	2.370(11)
Dy1-O2$^{\#2}$	2.431(5)	Dy1-O2$^{\#3}$	2.431(5)
Dy1-O1$^{\#2}$	2.386(6)	Dy1-O1$^{\#3}$	2.386(6)
O3$^{\#1}$-Dy1-O3	95.0(3)	O3- Dy1-O1W	80.2(3)
O3-Dy1-O2W	77.6(3)	O3$^{\#1}$-Dy1-O2$^{\#2}$	156.8(3)
O3-Dy1-O2$^{\#2}$	82.7(2)	O3-Dy1-O1$^{\#3}$	151.5(3)
O3-Dy1-O1$^{\#2}$	85.2(3)	O1W-Dy1-O2$^{\#2}$	121.8(2)
O1W-Dy1-O1$^{\#2}$	71.8(3)	O2W-Dy1-O1W	146.9(5)
O2W-Dy1-O2$^{\#2}$	79.4(3)	O2W-Dy1-O1$^{\#2}$	129.7(2)
O1$^{\#2}$-Dy1-O2$^{\#2}$	51.5(2)	O1$^{\#3}$-Dy1-O2$^{\#3}$	51.5(2)
O1$^{\#2}$-Dy1-O2$^{\#3}$	107.7(3)	O1$^{\#3}$-Dy1-O1$^{\#2}$	81.6(5)
		**2**	
Er1-O3	2.231(3)	Er1-O3$^{\#1}$	2.231(3)
Er1-O2W	2.323(6)	Er1-O1W	2.384(6)
Er1-O2$^{\#4}$	2.412(3)	Er1-O2$^{\#5}$	2.412(3)
Er1-O1$^{\#4}$	2.357(4)	Er1-O1$^{\#5}$	2.357(4)
O3-Er1-O1W	78.79(19)	O3-Er1-O2W	79.40(18)
O2W-Er1-O1W	146.6(3)	O3-Er1-O3$^{\#1}$	97.6(2)
O3-Er1-O2$^{\#4}$	83.66(16)	O3-Er1-O2$^{\#5}$	156.41(19)
O3-Er1-O1$^{\#4}$	150.6(2)	O3-Er1-O1$^{\#4}$	85.9(2)
O1W-Er1-O2$^{\#4}$	124.24(14)	O2W-Er1-O2$^{\#4}$	77.68(15)
O2W-Er1-O1$^{\#4}$	129.73(15)	O1$^{\#2}$-Er1-O1W	73.23(18)

Symmetry codes: $^{\#1}$ x, −y, z; $^{\#2}$1/2−x, 1/2−y, −z; $^{\#3}$1/2−x, −1/2+y, −z; $^{\#4}$1/2−x, −1/2+y, −z; $^{\#5}$1/2−x, 1/2−y, −z.

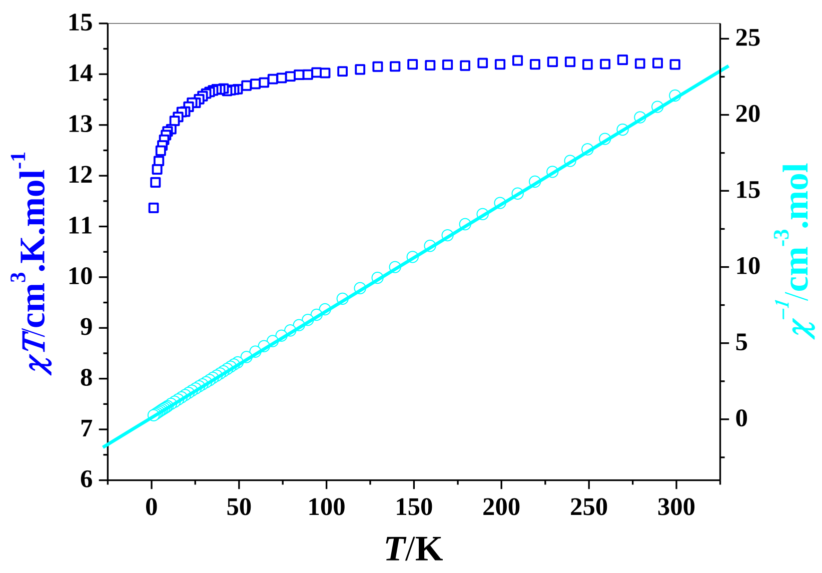

Fig. S2. Plots of χT vs T (□) and $1/\chi$ vs T (○) of complex **1**, the solid line represents the best theoretical fitting.

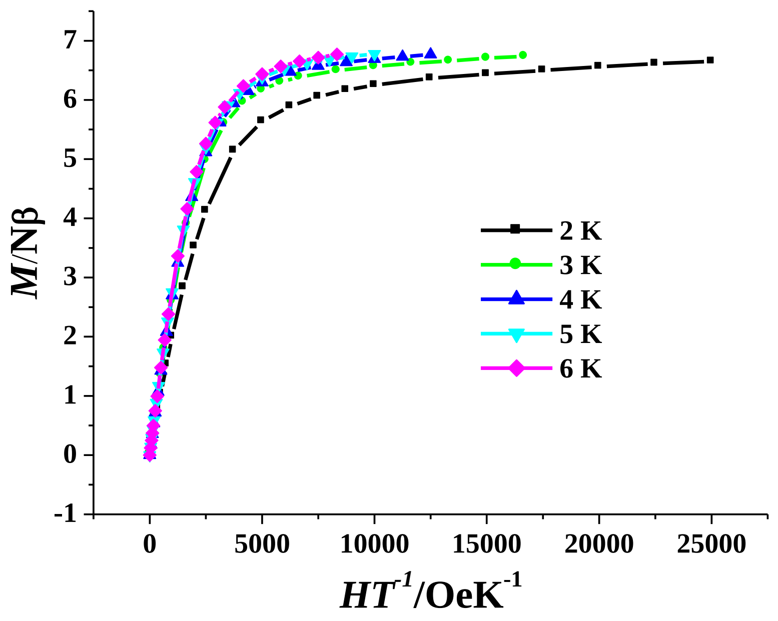

Fig. S3. M vs H/T plots at 2-6 K of **1**.

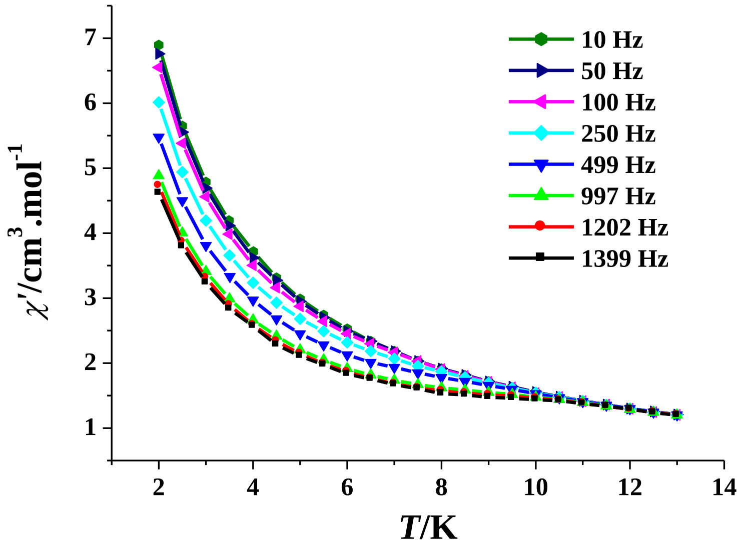

Fig. S4. Plots of χ' vs T for **1** (H_{dc} = 0 Oe, H_{ac} = 2.5 Oe).

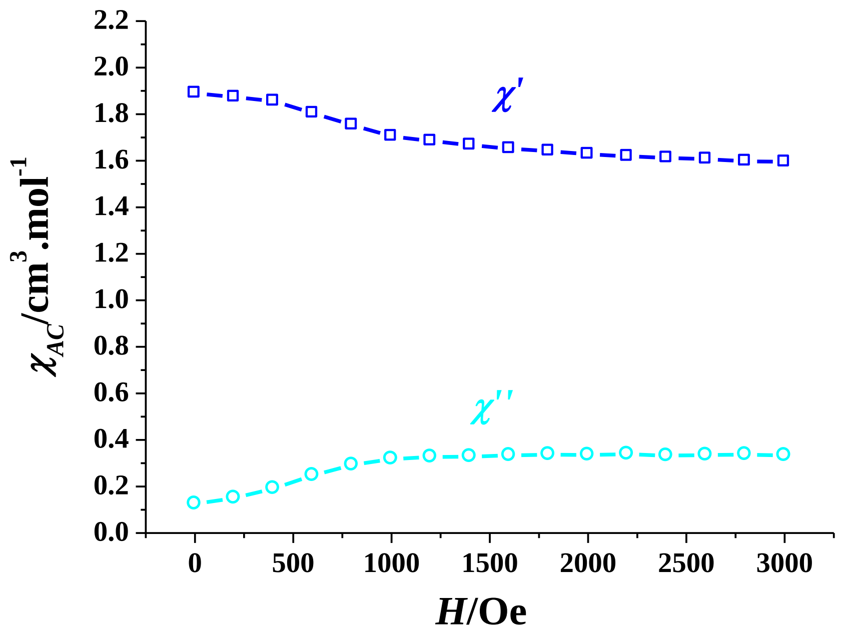

Fig. S5. AC susceptibilities measured in a 2.5 Oe ac magnetic field with variable dc fields at 250 Hz and at 7 K for **1**.

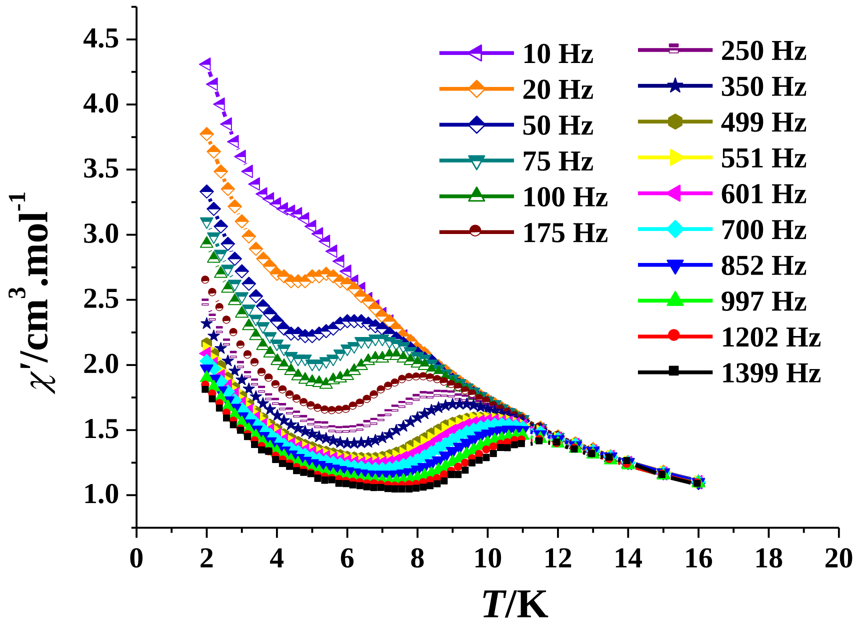

Fig. S6. Plots of χ' vs T for **1** (H_{dc} = 1 kOe, H_{ac} = 2.5 Oe).

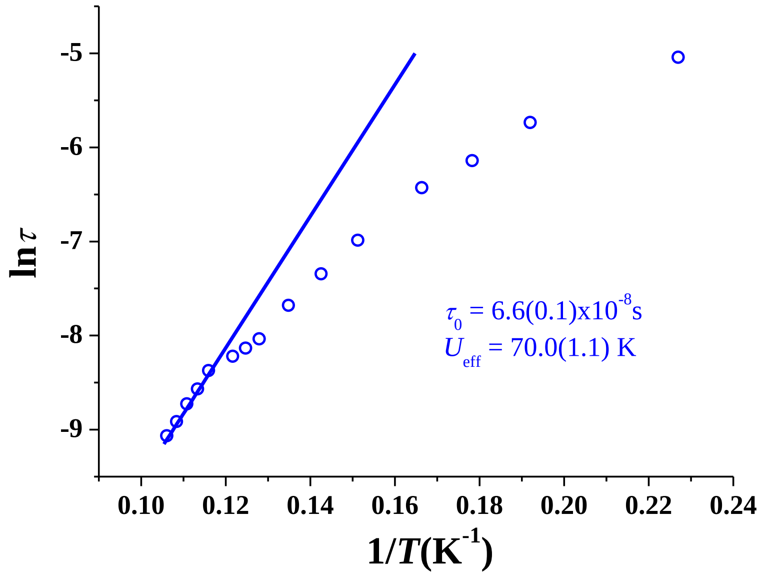

Fig. S7. Plot of ln(τ) vs 1/T for **1,** the solid line represents the best fitting with the Arrhénius law.

.

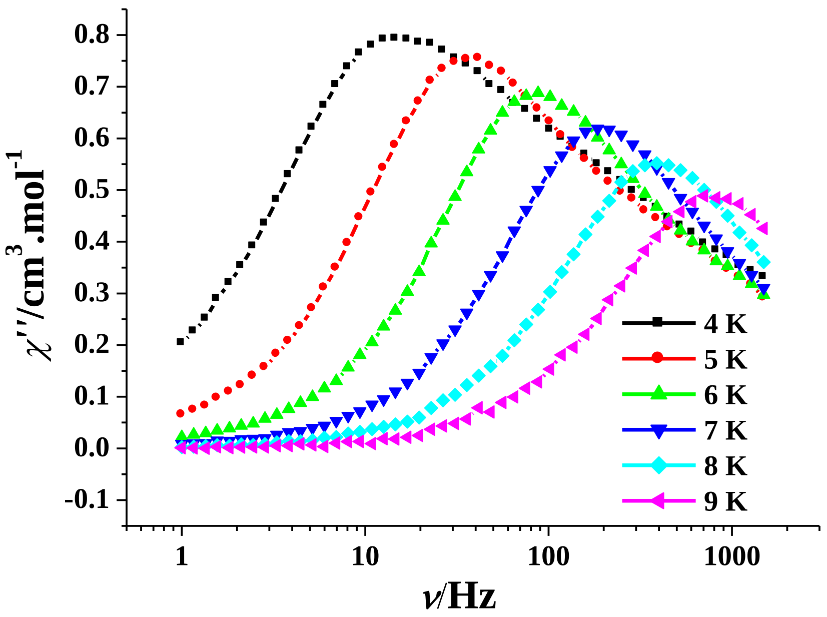

Fig. S8. Plots of χ'' vs ν for **1** (H_{dc} = 1 kOe, H_{ac} = 2.5 Oe).

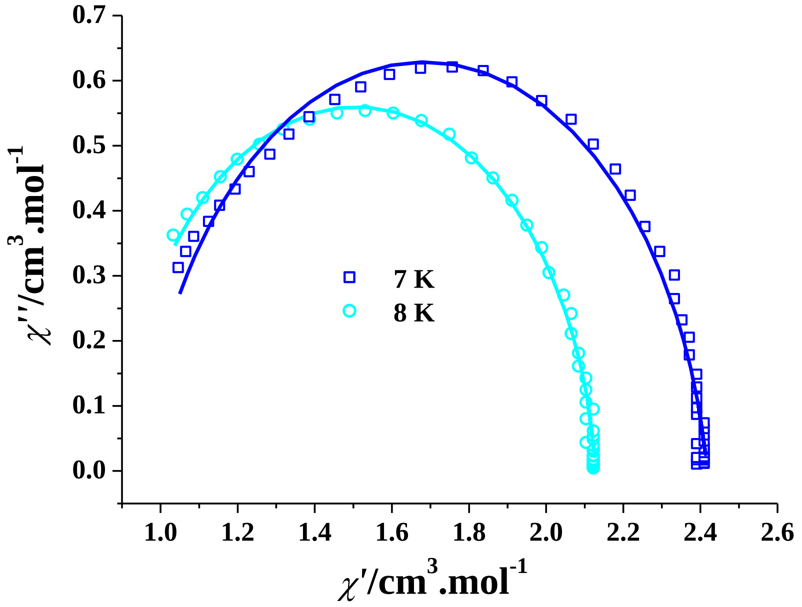

Fig. S9. Cole−Cole plots at 7 K and 8 K for **1** (H_{dc} = 1 kOe and H_{ac} = 2.5 Oe). The solid lines represent the best fitting

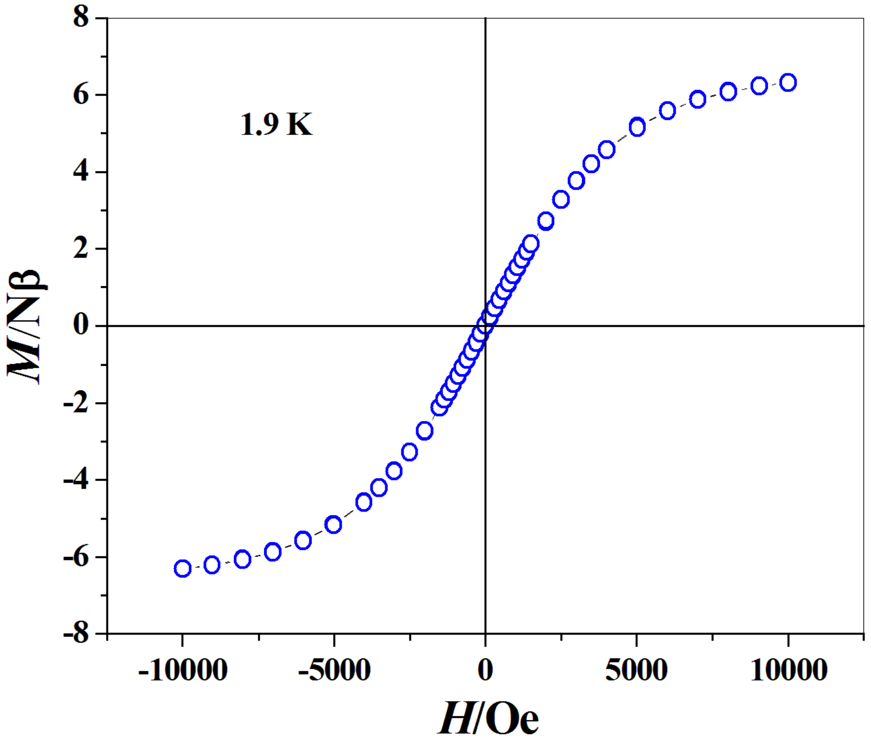

Fig. S10. Plot of *M* vs *H* at 1.9 K from −10000 to 10000 Oe for **1**.

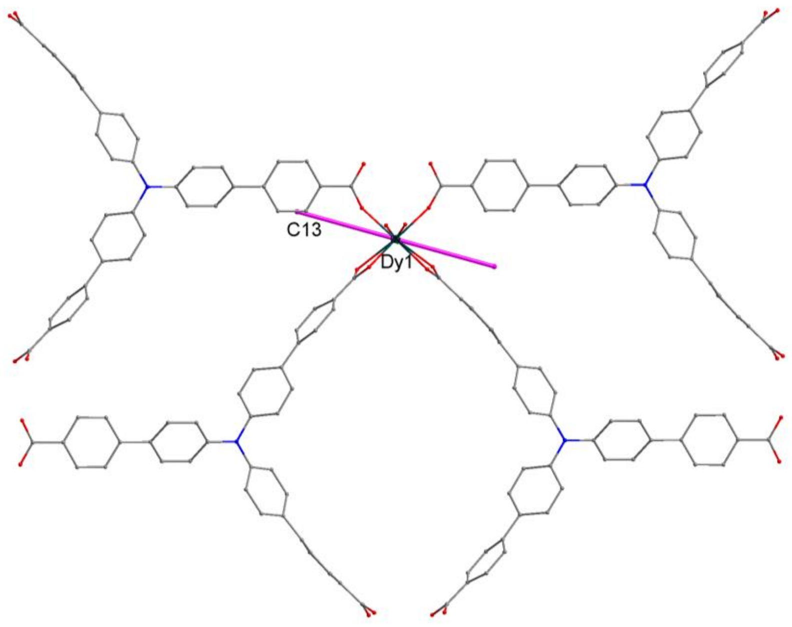

Fig. S11. Magnetic axis of the Dy^{3+} ion in **1** simulated by an electrostatic method.

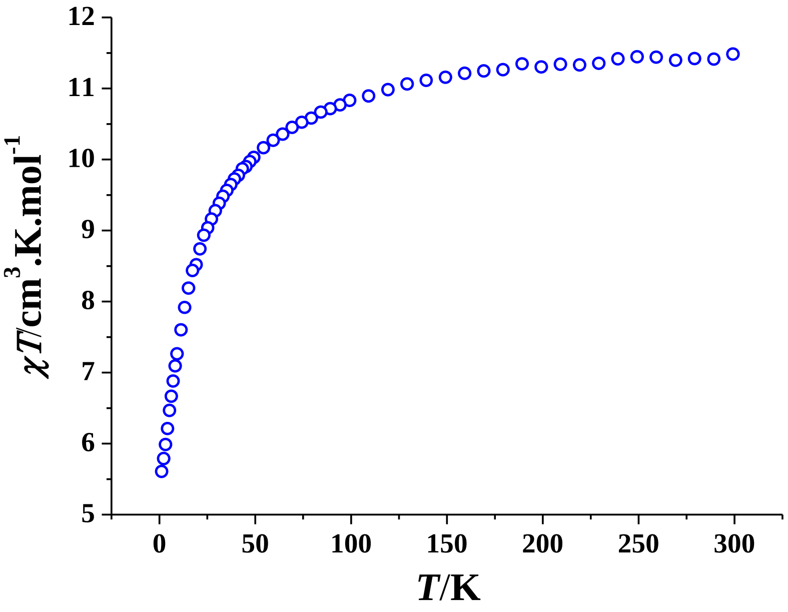

Fig. S12. Plot of χT as a function of T for **2.**

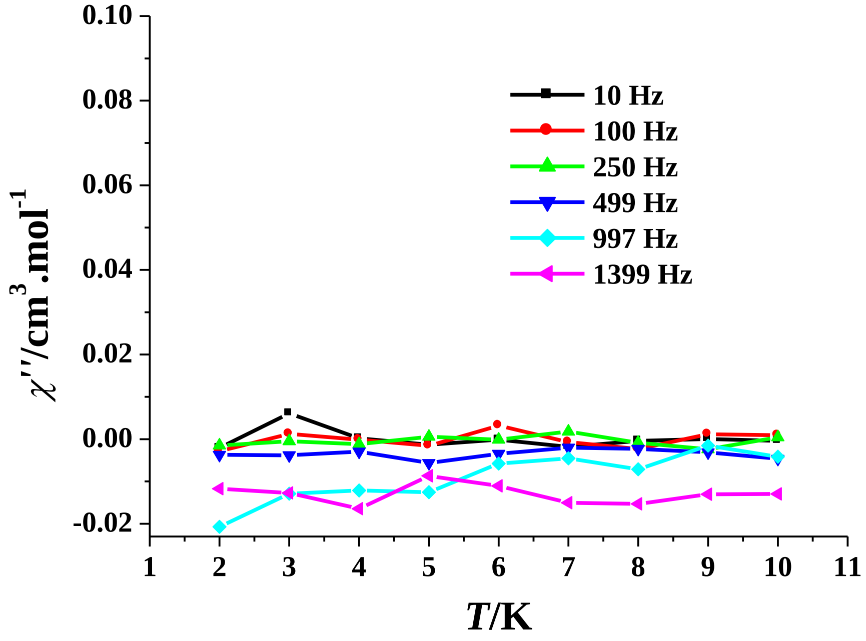

Fig. S13. Plots of χ'' vs T for **2** (H_{dc} = 0 Oe, H_{ac} = 2.5 Oe).

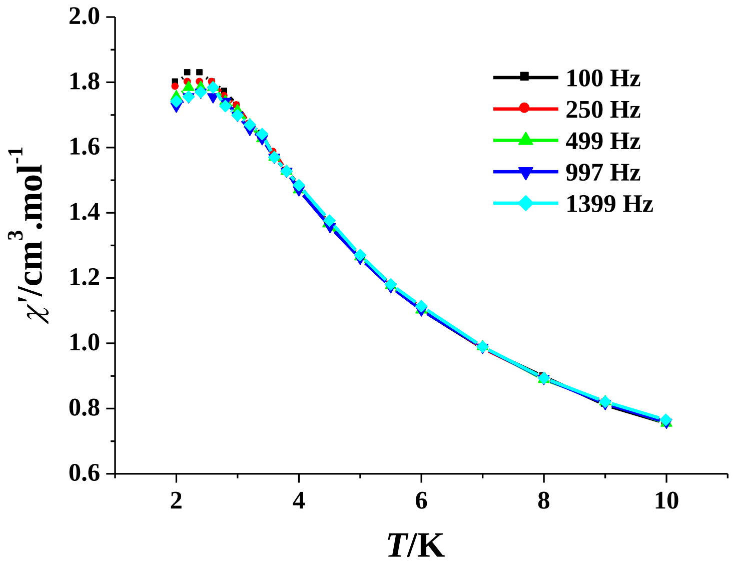

Fig. S14. Plots of χ' vs T for **2** (H_{dc} = 2 kOe, H_{ac} = 2.5 Oe).

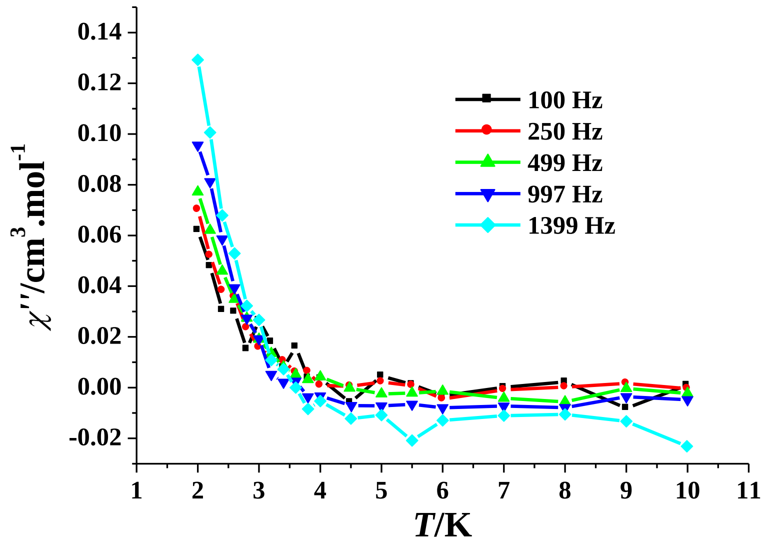

Fig. S15. Plots of χ'' vs T for **2** (H_{dc} = 2 kOe, H_{ac} = 2.5 Oe).

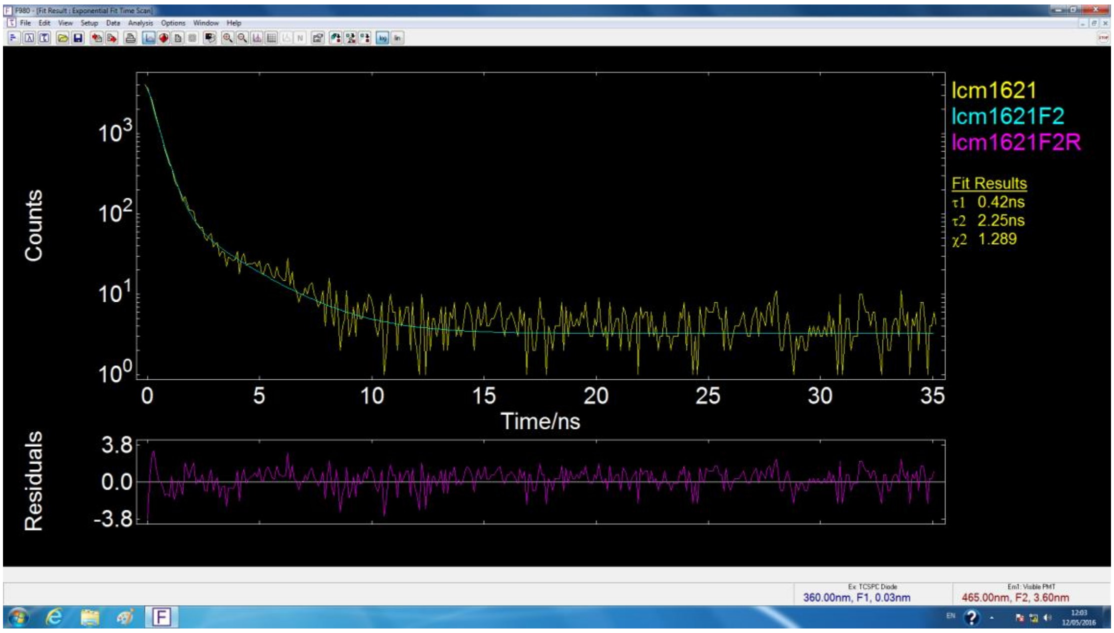

Fig. S16. The fluorescence decay and fitting curves for complex **1**, emission was monitored at 465 nm and the excitation was performed at 360 nm.

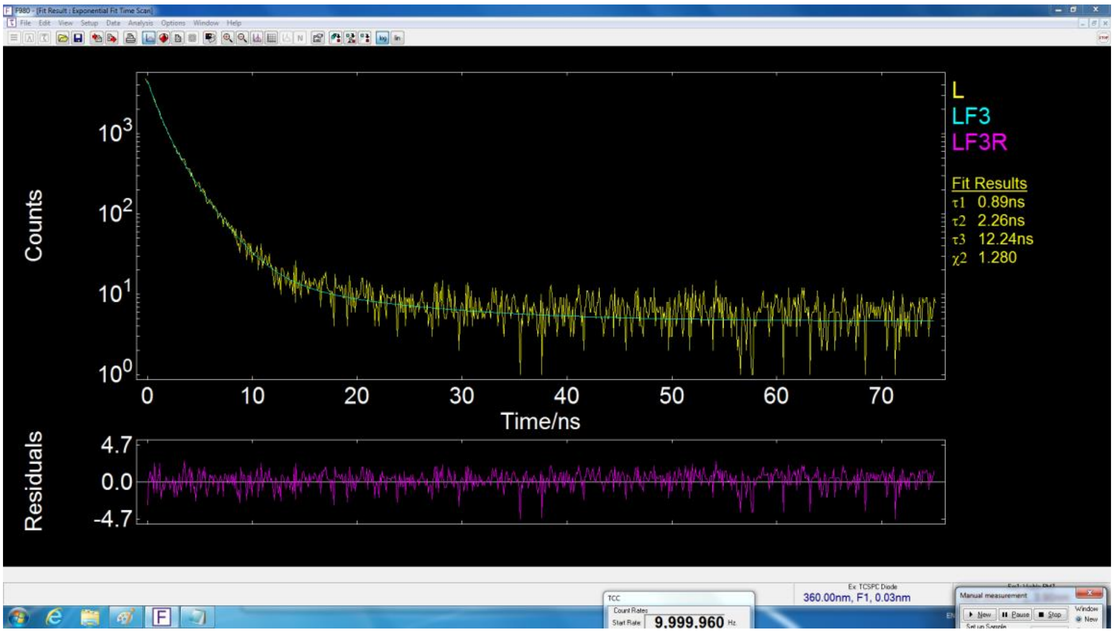

Fig. S17. The fluorescence decay and fitting curves for the ligand, emission was monitored at 498 nm and the excitation was performed at 360 nm.

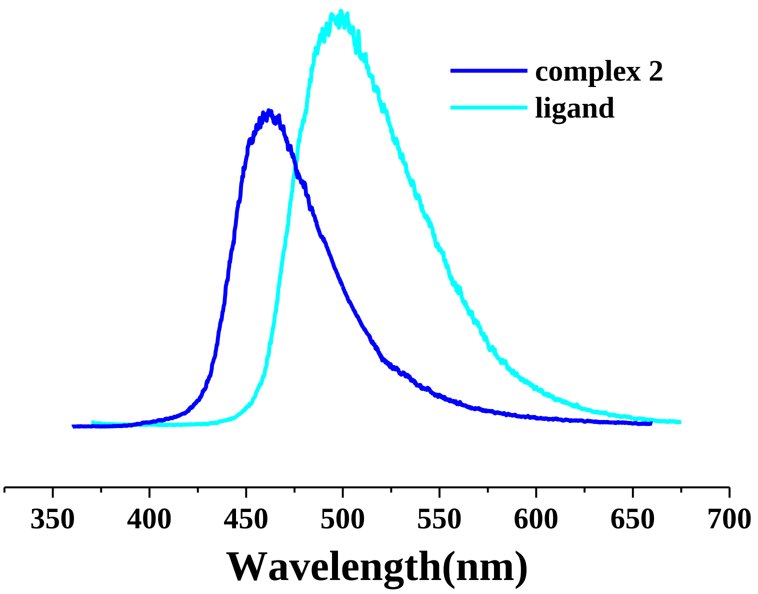

Fig. S18. Solid-state emission spectra of complex **2** and free ligand at room temperature (λ_{ex} = 350 nm).

Table S3. Crystal Data and Structural Refinement Parameters for 1 and 2.

	**1**	**2**
Chemical formula	$C_{39}H_{24}DyNO_8$	$C_{39}H_{24}ErNO_8$
Formula weight	797.09	801.85
Crystal system	Monoclinic	Monoclinic
Space group	*C2/m*	*C2/m*
a/Å	12.999(3)	13.316(3)
b/Å	40.569(8)	40.580(8)
c/Å	8.9856(18)	8.9240(18)
ß/ °	104.89(3)	104.18(3)
V/Å^3	4579.4(16)	4675.3(16)
Z	4	4
T/K	173 (2)	173 (2)
λ(Mo-Kα)/Å	0.71073	0.71073
ρ_{calc}/g · cm^{-3}	1.156	1.139
μ(Mo-Kα)/mm^{-1}	1.672	1.835
θ range	1.00°≤θ≤27.49°	1.00°≤θ≤27.50°
Limiting indices	−16≤h≤16,−52≤k≤52, −11≤l≤11	−17≤h≤16,−51≤k≤52, −11≤l≤11
Reflections collected	15996	16559
Unique reflections	5328	5451
$R_1{}^a$ [$I > 2\sigma(I)$]	0.0679	0.0522
$_wR_2{}^b$ [$I > 2\sigma(I)$]	0.2041	0.1441
$R_1{}^a$ [all data]	0.0761	0.0564
$_wR_2{}^b$ [all data]	0.2131	0.1478
S	1.085	1.046

$^aR_1 = \sum||Fo|-|Fc|| / \sum|Fo|$, $^b{}_wR_2 = \sum\{[w(Fo^2-Fc^2)^2] / \sum[wFo^2]^2\}^{1/2}$
